# Chromosomal Location and Identification of *TBX20* as a New Gene Responsible for Familial Bicuspid Aortic Valve

**DOI:** 10.3390/diagnostics15050600

**Published:** 2025-03-01

**Authors:** Yan-Jie Li, Su Zou, Yi-Zhe Bian, Xing-Yuan Liu, Chen-Xi Yang, Li Li, Xing-Biao Qiu, Ying-Jia Xu, Yi-Qing Yang, Ri-Tai Huang

**Affiliations:** 1 Department of Cardiology, Shanghai Chest Hospital, School of Medicine, Shanghai Jiao Tong University, Shanghai 200030, China; liyanjie6666@126.com (Y.-J.L.); qiuxingbiao@hotmail.com (X.-B.Q.); 2 Department of Cardiology, Shanghai Fifth People’s Hospital, Fudan University, Shanghai 200240, China; zou_su@163.com (S.Z.); voxens@outlook.com (Y.-Z.B.); 13472626672@163.com (C.-X.Y.); xuyingjia@5thhospital.com (Y.-J.X.); 3 Department of Pediatrics, Tongji Hospital, School of Medicine, Tongji University, Shanghai 200065, China; liuxingyuan402@tongji.edu.cn; 4 Key Laboratory of Arrhythmias, Ministry of Education of China, School of Medicine, Tongji University, Shanghai 200092, China; lilirz@tongji.edu.cn; 5 Cardiovascular Research Laboratory, Shanghai Fifth People’s Hospital, Fudan University, Shanghai 200240, China; 6 Central Laboratory, Shanghai Fifth People’s Hospital, Fudan University, Shanghai 200240, China; 7 Department of Cardiovascular Surgery, Renji Hospital, School of Medicine, Shanghai Jiao Tong University, Shanghai 200127, China

**Keywords:** congenital heart disease, congenital bicuspid aortic valve, human genetics, functional genomics, *TBX20*, biochemical assay

## Abstract

**Background/Objectives:** Congenital bicuspid aortic valve (BAV) signifies the most frequent category of congenital cardiovascular anomaly globally, occurring in approximately 0.5–2% of the general population worldwide. BAV is a major cause of thoracic aortopathy, encompassing aortic stenosis, aortic root dilation with regurgitation, aortic dissection, and aortic aneurysms, consequently leading to substantial late-onset morbidity and mortality. Accumulating evidence convincingly demonstrates the strong genetic basis underpinning BAV, though the inheritable reasons responsible for BAV in most patients remain largely obscure. **Methods:** A genome-wide genotyping with 400 polymorphic genetic markers followed by linkage analysis, haplotype assay, and sequencing analysis of candidate genes was conducted in a 4-generation BAV kindred of 47 individuals. Biochemical assays were performed to evaluate the functional effect of the identified mutation on TBX20. **Results:** A novel BAV-causative locus was mapped to chromosome 7p14. A sequencing assay of the genes within the mapped chromosomal region (locus) unveiled that only the c.656T>G (p.Ile219Arg) variation of *TBX20* was in co-segregation with BAV in the entire pedigree. The missense mutation was not uncovered in 322 healthy persons employed as control individuals. Functional deciphers revealed that the mutation significantly decreased the transcriptional activation of the representative target gene *ANP* and the binding ability to the *ANP* promoter and impaired the intranuclear distribution of TBX20. **Conclusions:** This investigation maps a new genetic locus (chromosome 7p14) linked to BAV and uncovers *TBX20* as a novel causative gene for familial BAV, adding more insight into the mechanisms underlying BAV and providing a molecular target for the individualized management of BAV.

## 1. Introduction

Congenital bicuspid aortic valve (BAV) signifies the most frequently occurring form of cardiovascular developmental malformation in humans worldwide, with an overall prevalence of approximately 0.5–2% in the general population globally [1,2]. Congenital BAV can occur as an isolated aberration or concur with other distinct types of congenital heart disease (CHD), such as atrial septal defect (ASD), aortic coarctation/stenosis, patent ductus arteriosus, pulmonary stenosis, ventricular septal defect, and endocardial cushion defect [1]. In patients with other types of CHD, the prevalence of BAV is much higher, as indicated by an example that in patients with severe aortic stenosis requiring surgery, the prevalence of BAV is as high as 50% [2]. Although BAV is frequently diagnosed by echocardiographic imaging in asymptomatic patients, approximately 33% of BAV patients suffer from severe thoracic aortic complications/valvulo-aortopathy during their lifespan, including aortic valve stenosis, aortic dissection, aortic root dilation with regurgitation, and aortic aneurysm, as well as bacterial endocarditis, cardioembolic cerebral stroke, ventricular fibrosis, hypoplastic left ventricle, retinal infarction, cardiac dysrhythmias, heart failure, and, even, premature cardiac demise [1,3,4,5,6,7,8,9,10]. It has been reported that BAV is accountable for aortic stenosis in about 70% to 85% of pediatric patients and over 50% of adult patients [10]. Moreover, aortic dilatation is detected in 30% to 70% of BAV patients, and a dysfunctional aortic valve is observed in up to 47% of BAV cases [10]. BAV also confers an 8-fold enhanced risk for aortic dissection, and the BAV-increased risks for aortic aneurysm occurrence, aortic valve replacement, and aortic surgery are 26%, 53%, and 25%, respectively, over 25 years [10]. It was reported that in the US in 2017, aortic valve disease was accountable for about 60% of the overall deaths caused by valvular heart disease [11]. At present, the trans-catheter interventional or surgical replacement of aortic valves remains the only durable treatment option, with no effective pharmacological therapies available for BAV [11,12]. Consequently, BAV leads to substantial late-onset/long-term morbidity, as well as mortality, posing an enormous socioeconomic encumbrance [11,13,14,15]. Despite the pronounced clinical importance, the causes leading to BAV and the pathological mechanisms underpinning BAV remain largely elusive.

During embryogenesis, the heart begins to form as a single heart tube comprising an outer layer of the myocardium and an inner lining of the endocardium [16]. These two layers of the heart tube are separated by cardiac jelly, a thick hydrophilic region produced by cardiomyocytes, full of extracellular matrix, proteoglycans, glycosaminoglycans, and hyaluronic acid [16]. In the cardiac jelly, the primitive cardiomyocytes secrete some factors that drive the transition of endothelial cells into mesenchymal cells (a biological process known as endothelial-to-mesenchymal transition, namely EMT), resulting in the invasion of mesenchymal cells into the cardiac jelly [16]. In the cardiac outflow tract, EMT contributes to the formation of a parietal and a septal cushion, the primordia of the semilunar valves (aorta/pulmonary artery valves), and the septum of the outflow tract [16]. The other sources of mesenchymal cells, which are also involved in populating the endocardial cushions, originate from neural crest cells, epicardial cells, and the cells stemming from the second heart field (SHF) [1,16]. In the aortic valve, the parietal cushion (endothelial/neural crest-originated) develops into the right coronary cusp/leaflet; the septal cushion (endothelial/neural crest-originated) gives rise to the left coronary cusp/leaflet, while the non-coronary cusp/leaflet derives solely from the intercalated cushion (endothelial/neural crest/SHF-originated) on the posterior side of the outflow tract [1,16]. The normal development of endocardial cushions, the precursors of mature heart valves, is essential for proper valvular morphogenesis, and so either a failure of EMT or a defect in the contributing cell lineages (especially in cellular proliferation, specification, differentiation, migration, adhesion, and apoptosis) may lead to aberrant aortic valvulogenesis and the pathogenesis of BAV (the abnormal fusion of two aortic valve leaflets), mainly due to decreased mesenchyme populations in the endocardial cushions [1,10,11,16].

Now, it is generally understood that normal aortic valvulogenesis undergoes a complicated biological process, including the formation and elongation of the endocardial cushion and the structural remodeling of the valvular cusps or leaflets, and any pathogenic factor that disturbs this complex developmental process may result in abnormal aortic valvular morphogenesis, including BAV [1,10,11,16,17]. Aggregating genetic investigations have demonstrated that congenital BAV is an inherited disorder, with a prevalence of roughly 9% in the first-degree relatives of BAV patients and up to 24% in those of larger BAV families [1,10,11,18]. In addition to genomic copy number variants [19], deleterious variations in an increasing number of genes have been implicated in the pathogenesis of BAV [1,10,11,17]. To date, there has been convincing cumulative evidence that pathogenic variations in >20 genes each may give rise to a small percentage of BAV, encompassing those encoding cardiac transcription factors (GATA4-6, TBX5, NKX2.5, HOXA1, KLF13, and NR2F2), signaling molecules (NOTCH1, SMAD4, and SMAD6), extracellular matrix proteins (FBN1 and FBN2), collagen proteins (COL1A2 and COL1A1), metalloproteinases (MMP9, ADAMTS5, ADAMTS16, and ADAMTS19), E3 ubiquitin ligase (MIB1), filamin A (FLNA), roundabout receptor proteins (ROBO4 and ROBO1), myosin heavy chain 6 (MYH6), mucin 4 (MUC4), palmdelphin (PALMD), protocadherin alpha 9 (PCDHA9), and cilia proteins (EXOC8, EXOC6, and EXOC4) [1,10,11,17,20,21,22,23,24]. Nevertheless, rare genetic variants in these established BAV-causative genes can merely explain a tiny minority of BAV patients, and the defective genetic components accountable for the development of BAV remain to be identified in most BAV-affected individuals.

## 2. Materials and Methods

### 2.1. Enrollment and Clinical Examination of Research Participants

For this clinical investigation, a 47-member pedigree (specifically designated as Family BAV-1) spanning four generations affected with congenital BAV was discovered in a Chinese population of Han ethnicity. The proband (Family BAV-1: II-1) and the available family members who were referred to for cardiac evaluation during the period from 12 October 2016 to 29 March 2019 were recruited. In total, 322 unrelated healthy individuals with no family history of CHD were enrolled as control persons. The control people were randomly selected from the volunteers who completed a routine physical examination and had no CHD (including BAV) between October 2016 and June 2021. A detailed clinical investigation of the study participants was conducted by experienced cardiologists and echocardiographers, encompassing comprehensive reviews of their personal/familial histories (a minimum of 3 generations) and medical histories (including CHD diagnosed previously, trans-catheter interventional procedures, and surgical operations), comprehensive and careful physical examinations, routine laboratory tests, and transthoracic echocardiograms with color Doppler. When indicated, a contrast-enhanced cardiac computed tomographic angiography was performed. A diagnosis of BAV was made if two distinct aortic valvular leaflets were confirmed by 2-dimensional echocardiographic images and/or by the medical records of trans-catheter interventional or surgical treatments of BAV-related aortopathies [10,17]. The current research was fulfilled in compliance with the standards summarized in the Helsinki Declaration released in 1975, as revised in 2008. The research protocols used for the current investigation were approved by the medical ethics committees of the Tongji Hospital, Tongji University (ethical approval code: LL(H)-09-07), and the Shanghai Fifth People’s Hospital, Fudan University (ethical approval number: 2020-086), according to the local ethical regulations and the relevant national guidelines on human research. An informed consent form was signed by the research subjects or their parents/legal guardians before recruiting the study participants for this study. Peripheral blood specimens were collected in acid–citrate–dextrose (ACD)-anti-coagulated tubes from each research subject to extract genomic DNA (gDNA).

### 2.2. Whole-Genome Screening with Microsatellite Markers Followed by Linkage Assay

A pan-genome scan and genotyping were implemented in the 44 living members of Family BAV-1 affected with BAV by utilizing a linkage mapping set (version 2.5; Applied Biosystems, Foster City, CA, USA), which contains a total of 400 highly polymorphic microsatellite markers (short tandem repeats), spaced across the human genome at an even density of ~9.2 cM, as described elsewhere [25,26,27]. With the fluorescently labeled primers, multiplex amplification of the markers-containing gDNA fragments was conducted via polymerase chain reaction (PCR) using a Taq DNA Polymerase kit (Applied Biosystems, Foster City, CA, USA) on a thermal cycler instrument (Bio-Rad, Hercules, CA, USA). The amplicons were separated through gel electrophoresis on a DNA analysis apparatus (Applied Biosystems, Foster City, CA, USA), following the user’s manual. Allele calling was conducted with GeneMapper (version 3.1; Applied Biosystems, Foster City, CA, USA). To search for a locus linked to BAV in the family, a linkage analysis was carried out in all genotyped family members to obtain the two-point logarithm of odds (LOD) scores for every microsatellite marker, as previously described [25,26,27]. When a 2-point LOD score (a LOD score ≥ 2.0) supportive of linkage to BAV was yielded for a microsatellite marker, several additional markers around the marker with evidence for linkage were applied to finely map the locus. Haplotypes of Family BAV-1 affected with BAV were constructed to display the shared chromosomal region among the pedigree members suffering from BAV and confine the recombinant borders of a chromosomal region.

### 2.3. Sequencing Assay of the Genes Within the Located BAV Locus

As previously described [28,29,30,31], a whole-exome sequencing (WES) analysis was conducted in two members suffering from BAV (Family BAV-1: II-1 and III-2), as well as one unaffected member (Family BAV-1: III-3). In short, the gDNA samples from the three chosen family members of Family BAV-1 underwent exome capture with a targeted capture kit, the SureSelect Human All Exon V6 kit (Agilent Technologies, Santa Clara, CA, USA), following the manufacturer’s recommendations. The captured DNA was subjected to WES under the HiSeq4000 platform (Illumina, San Diego, CA, USA), following the user’s manual. Read data were aligned to the referential human genome (build GRCh37/hg19) with the BWA (version 0.7.17) tool [32]. With the GATK (version 3.8.1) software [33], DNA sequence variations (single-nucleotide variations and small deletions/insertions) at the targeted regions of individual genomes were identified. The pathogenic effect of all variants was predicted with the VEP (version 99) toolset [34] and annotated with ANNOVAR (version 2015) [27]. The non-synonymous variations and splicing donor/acceptor variations underwent further analysis, including the Sanger sequencing assay of a gene harboring a pathogenic variation and a co-segregation assay in the whole BAV pedigree (Family BAV-1). After a gene carrying a BAV-causative mutation was identified in Family BAV-1, sequence assays of the same gene were completed in 322 unrelated healthy people. For a verified deleterious variation, databases of population genetics, such as SNP and gnomAD, were queried to verify its novelty, as described elsewhere [27].

### 2.4. Preparation of Gene-Expressing Plasmids

The plasmid of TBX20-pcDNA3.1 was generated as described previously [27]. The Ile219Arg-mutant TBX20-pcDNA3.1 expression vector was yielded by mutagenesis of TBX20-pcDNA3.1 using a site-targeted mutagenesis kit (Invitrogen, Carlsbad, CA, USA), as well as a pair of primers (5′-ATCAACATGGCCATAGAATTTTGAACTCAAT-3′ and 5′-ATTGAGTTCAAAATTCTATGGCCATGTTGAT-3′), and then it was validated by a direct sequencing analysis. The promoter of the human atrial natriuretic peptide (ANP)-encoding gene (GenBank accession number: NC_000001.11), a 960-bp gDNA fragment (from −930 to +30, with the transcriptional start site designated as +1) of the human ANP gene harboring multiple consensus TBX20-binding sites, was PCR-amplified from human gDNA utilizing Platinum^TM^ Taq DNA Polymerase (Invitrogen, Carlsbad, CA, USA) and a specific pair of primers (5′-GCAGCTAGCTGAGGGAAACTGACTATACC-3′ and 5′-TGCCTCGAGCCTCTCTTGGCCTACGTCTG-3′). The amplicons were doubly cut with the endonucleases *Nhe*I and *Xho*I and ligated with the pGL3-Basic vector (Promega, Madison, WI, USA) utilizing T_4_ DNA ligase to produce the *ANP* promotor-driven Firefly luciferase expression vector (ANP-luc).

### 2.5. Cellular Transfection with Expression Plasmids and Dual-Luciferase Activity Measurement

COS7 cells were routinely cultured and seeded into the wells of a 24-well plate 24 h before transient transfection. The cells were transfected with various expression plasmids utilizing the Lipofectamine^TM^ 3000 Transfection Reagent (Invitrogen, Carlsbad, CA, USA). To evaluate the transactivation of *ANP* by TBX20, the COS7 cells were transfected with 800 ng of empty pcDNA3.1 (−) or 800 ng of wild-type TBX20-pcDNA3.1 (TBX20), 800 ng of Ile219Arg-mutant TBX20-pcDNA3.1 (Ile219Arg), 400 ng of TBX20 plus 400 ng of (−), or 400 ng of TBX20, plus 400 ng of Ile219Arg, in the presence of 1000 ng of ANP-luc and 20 ng of pRL-TK expressing Renilla luciferase (Promega, Madison, WI, USA). Here, the pRL-TK vector was used as an internal control to normalize the transfection efficiency. The cells were collected and lysed 48 h post-transfection. The dual-luciferase activities of cellular lysates were quantitatively measured, and the ratio of Firefly luciferase activity to Renilla luciferase activity was utilized to measure the promoter activity of the target gene *ANP*, as described previously [27,35,36,37].

### 2.6. DNA-Binding Ability of Wild-Type and Ile219Arg-Mutant TBX20 Proteins

The collection of COS7 cells was completed 48 h after transient transfection, and nuclear proteins were prepared as described elsewhere [27]. The DNA-binding ability of the TBX20 protein was studied by an electrophoretic mobility shift assay with the *ANP* probe. The oligonucleotides of the *ANP* probe with a TBX20-binding site were synthesized and labeled with biotin on the 5′ end. The *ANP* probes (0.2 pmol) and purified wild-type or Ile219Arg-mutant TBX20 nuclear protein were incubated in binding buffer (Beyotime Biotechnology, Shanghai, China) for 20 min. Parallel unlabeled cold probes (20 pmol) were preincubated with purified wild-type or Ile219Arg-mutant TBX20 nuclear protein for 10 min. Both the labeled probe and unlabeled cold probe are the wild-type DNA sequences located in the promoter of *ANP*. Electrophoresis was performed to fractionate the protein–DNA complexes on 6% nondenaturing polyacrylamide gels at a voltage of 100 V for one hour. Then, the protein–DNA complexes were transferred to a positively charged nylon membrane (ThermoFisher Scientific, Waltham, MA, USA) at 380 mA for 30 min and cross-linked by exposure to ultraviolet for 20 min. The interaction of TBX20 with ANP promoter DNA was probed using streptavidin-horseradish peroxidase conjugates and detected with an EMSA kit (Beyotime Biotechnology, Shanghai, China).

### 2.7. Subcellular Distribution of Wild-Type and Ile219Arg-Mutant TBX20 Proteins

COS7 cells were cultivated on coverslips in a 12-well plate. When the cells reached an 80% growth density on the culture plate, the COS7 cells were transfected with 200 ng of empty pcDNA3.1, wild-type TBX20-pcDNA3.1, or Ile219Arg-mutant TBX20-pcDNA3.1. The cells were fixed 48 h after transfection using 4% paraformaldehyde and rinsed briefly in cooled phosphate buffer saline (PBS). The fixed COS7 cells were heated at 95 °C in an antigen retrieval solution. Next, the cells were permeabilized utilizing 0.5% Triton X-100 for 5 min and blockaded with 3% bovine serum albumin (BSA) for 30 min. Subsequently, coverslips were blocked using anti-TBX20 polyclonal antibody (ABclonal, Wuhan, Hubei Province, China) at a 1:100 dilution in an immunohistochemical wet box overnight at 4 °C. The cells were washed with PBS-Tween three times and immunostained using goat anti-rabbit Alexa-Flour 594-conjugated secondary antibody (Abcam, Shanghai, China) in the immunohistochemical wet box. The cellular nuclei were stained using 4′,6-diamidino-2-phenlindole (DAPI; Sigma-Aldrich, St. Louis, MO, USA). Images were obtained under a confocal laser-scanning fluorescence microscope (Leica Microsystems, Mannheim, Germany).

### 2.8. Statistical Analysis

A statistical analysis was performed, as previously described [27]. In brief, the continuous data for the promoter activities were given as mean values with standard deviations (SDs). A student’s unpaired t-test was applied when the comparison was performed between two groups. For the comparison among three or more groups, the continuous variables were analyzed by utilizing a one-way analysis of variance, followed by Tukey’s post-hoc test. A 2-tailed *p*-value < 0.05 indicated a significant difference.

## 3. Results

### 3.1. Clinical Characteristics of the Study Pedigree with BAV

**Figure 1:**
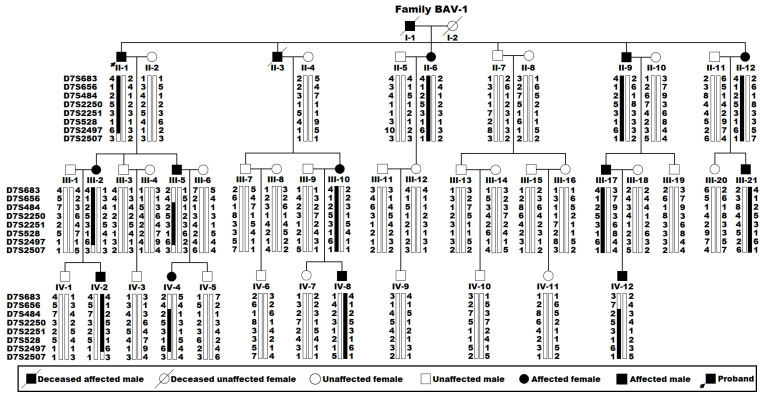
Haplotype analysis of a multigenerational pedigree segregating bicuspid aortic valve (BAV). An individual pedigree member is recognized by Roman–Arabic numbers. Individual haplotypes around the *TBX20* locus were indicated by listing the allelic genotypes (symbolized with Arabic numerals) for the chosen polymorphic genetic markers D7S683, D7S656, D7S484, D7S2250, D7S2251, D7S528, D7S2497, and D7S2507 (from top to bottom) by each individual. A vertical bar signifies the chromosomal region confined by genotype assays utilizing the selected eight markers. Blackened vertical bars indicate haplotypes segregating BAV, while open ones denote normal haplotypes. Chromosomal recombinant events in individuals II-1, III-5, and IV-12 are defined by the BAV locus on chromosome 7p14.1-p14.3, a chromosomal segment between marker D7S656 and marker D7S2507.

As displayed in Figure 1, a 47-member pedigree across four generations with BAV (Family BAV-1) was identified, from which 44 living pedigree members were recruited, clinically investigated, and genotyped. Among the 44 living pedigree members, 13 had congenital BAV, encompassing 8 male members and 5 female members, with an average age of 37.77 ± 19.72 years, ranging between 10 and 69 years.

**Figure 2:**
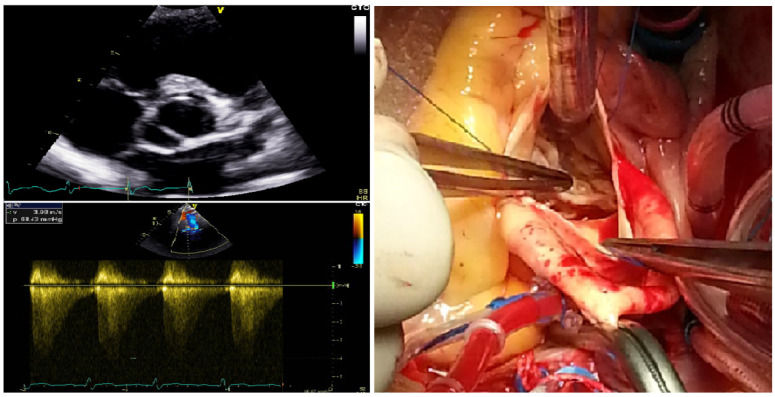
A representative two-dimensional echocardiogram, a color Doppler echocardiogram, and a surgical view photography of the index patient (II-1) from Family BAV-1. The representative two-dimensional echocardiogram (up left), color Doppler echocardiogram (down left), and surgical view photograph (right) illustrate the bicuspid aortic valve and aortic valve stenosis.

In this study pedigree (Family BAV-1), there was a total of 15 family members affected with BAV documented on their echocardiograms, including 13 living family members, as shown in Table 1. The proband (Family BAV-1: II-1) was diagnosed with congenital BAV (fusion of the left and right cusps) and severe aortic valve stenosis and underwent surgical replacement of aortic valves. A representative two-dimensional echocardiogram, a color Doppler echocardiogram, and a surgical view photograph of the index patient are given in Figure 2.

The proband’s father (Family BAV-1: I-1) and younger brother (Family BAV-1: II-3) were both diagnosed with congenital BAV (fusion of the left and right cusps) and aortic valve stenosis and died of acute rupture of the aortic aneurysm and aortic dissection, respectively. Among the 13 pedigree members with congenital BAV, 3 (Family BAV-1: II-9, III-17, and IV-12) also had congenital ASD. The proband’s two younger brothers (Family BAV-1: II-6, II-9) also had aortic valve stenosis. The clinical characteristic profiles and the *TBX20* genotypes of the living pedigree members affected with congenital BAV from Family BAV-1 are provided in Table 1.

Additionally, a group of 322 unrelated healthy persons with no family history of CHD (197 male and 125 female persons, with a mean age of 38.16 ± 12.07 years), who were enrolled from the general population of Han ethnicity in China, were employed as control individuals. The clinical investigation revealed that all 322 control subjects had a normal cardiac structure with no BAV, as shown in their color Doppler echocardiograms.

### 3.2. A Novel Human BAV-Linked Locus on Chromosome 7p14

A whole-genome screening with genetic markers and a genetic linkage assay were completed in Family BAV-1 with congenital BAV for the strictly defined phenotype (congenital BAV alone). In the linkage analysis for the phenotype of congenital BAV, the maximum two-point LOD score (Zmax) of 5.2802 at no recombination (recombination fraction θ = 0.00) was preliminarily obtained at marker D7S484 on chromosome 7p14, providing convincing evidence of significant linkage. To refine the BAV locus, seven additional genetic markers (D7S683, D7S656, D7S2250, D7S2251, D7S528, D7S2497, and D7S2507) at the nearby chromosomal loci surrounding marker D7S484 were applied to genotype the living members from Family BAV-1, with a Zmax of 5.4185 at θ = 0.00 for markers D7S2250 and D7S2497, and the BAV haplotype of Family BAV-1 was deduced using the eight genetic markers (Figure 1). Recombination events took place in one BAV-affected individual (Family BAV-1: II-1) at marker D7S2507 and two BAV-affected individuals (Family BAV-1: III-5 and IV-12) at markers D7S683 and D7S656, which defined a critical chromosomal interval for BAV, a novel familial BAV-causative locus, on chromosome 7p14 (7p14.1–p14.3; GRCh38.p14, chr7: 34,158,089–38,860,523), a ~4.62 cM (~4.70 Mb) chromosomal interval confined by markers D7S656 and D7S2507. The LOD scores for the chosen eight markers applied to generate the haplotype of Family BAV-1 are provided in Table 2.

### 3.3. Identification of TBX20 as a Novel Familial BAV-Causing Gene

**Figure 3:**
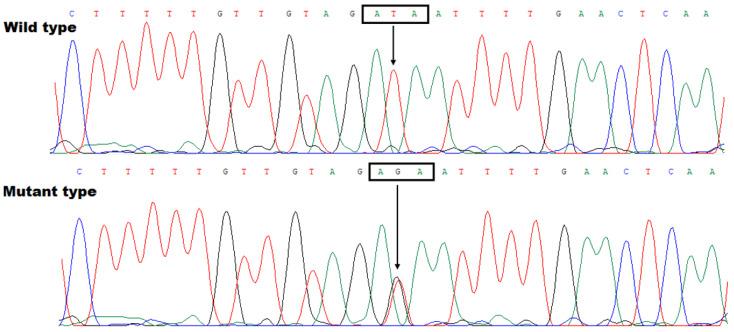
Sequence electropherograms exhibiting the *TBX20* c.656T>G mutation in a heterozygous status along with its control counterpart in a homozygous status. A vertical arrow points down to T/G from the proband of Family BAV-1 (mutant type) or T/T from an unaffected member of Family BAV-1 (wild-type). The three nucleotides within the rectangle constitute a coding codon.

As listed in Table A1, there are 87 known genes at the mapped chromosomal locus (a 4.70 Mb region) between D7S656 and D7S2507, including 18 genes coding for protein, 21 genes coding for non-coding RNAs, and 48 pseudogenes. Via WES and a bioinformatical analysis in two pedigree members affected with BAV (Family BAV-1: II-1 and III-2) and one unaffected pedigree member (Family BAV-1: III-3), followed by a Sanger sequencing analysis of *TBX20* (including all exons and splicing donors/acceptors), in all the available members from Family BAV-1, we discovered that merely the mutation c.656>;p. (Ile219Arg) of *TBX20* (NM_001077653.2) was linked to/in co-segregation with the BAV phenotype in the whole pedigree. There are no other protein-altering variants present in the 4.70 Mb region that co-segregate with the disease in Family BAV-1. The discovered *TBX20* missense mutation was neither observed in a cohort of 322 unrelated healthy people nor found in the databases of SNP and gnomAD, indicating a new mutation. The sequence electropherograms exhibiting the *TBX20* c.656T>G mutation in a heterozygous status along with its control counterpart in a homozygous status are illustrated in Figure 3.

### 3.4. Significantly Decreased Transactivation of ANP by Ile219Arg-Mutant TBX20

**Figure 4:**
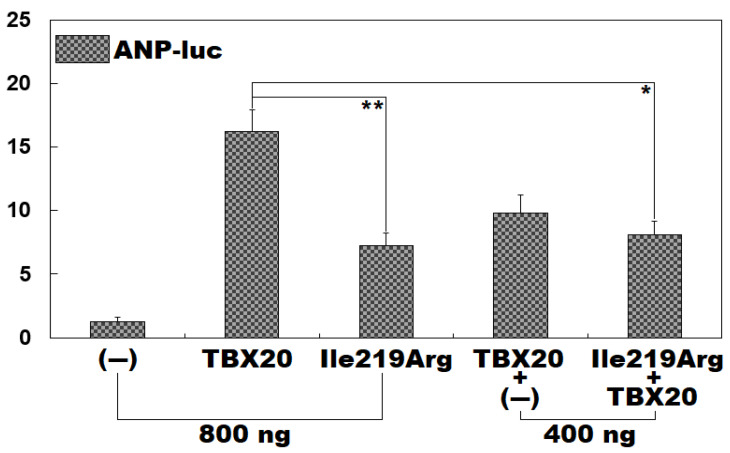
Reduced transactivation of *ANP* by Ile219Arg-mutant TBX20. In vitro in cultivated COS7 cells transfected with various expression vectors, a quantitative dual-luciferase activity measurement indicated that Ile219Arg-mutant TBX20 conferred a significantly diminished transactivation of *ANP*. A dual-reporter gene measurement was implemented three times in triplicate for each expression vector. Here, ** indicates *p* < 0.005 and * indicates *p* < 0.01 in comparison with TBX20 (800 ng).

As displayed in Figure 4, in contrast to the wild-type TBX20 (TBX20), the Ile219Arg-mutant TBX20 (Ile219Arg) had a significantly diminished transactivation of the target gene *ANP* either in a homozygous status or in a heterozygous status. Specifically, in a homozygous status, TBX20 (800 ng) and Ile219Arg (800 ng) transactivated *ANP* by ~16-fold and ~7-fold, respectively (TBX20 vs. Ile219Arg: t = 8.0182, *p* = 0.0013), while, in the heterozygous status, 400 ng of TBX20 plus 400 ng of Ile219Arg transcriptionally activated *ANP* by ~8-fold (TBX20 vs. Ile219Arg + TBX20: t = 7.0462, *p* = 0.0059). Similar results were generated (F = 60.9672, *p* = 5.503 × 10^−7^) when the comparison was conducted among multiple groups. Specifically, for (−) vs. TBX20: t = 15.0167, *p* = 2.228 × 10^−7^; for (−) vs. Ile219Arg: t = 5.9867, *p* = 0.0008; for (−) vs. TBX20 + (−): t = 8.5700, *p* < 0.0001; for (−) vs. Ile219Arg + TBX20: t = 6.8533, *p* = 0.0003; for TBX20 vs. Ile219Arg: t = 9.0300, *p* = < 0.0001; for TBX20 vs. TBX20 + (−): t = 6.4467, *p* = 0.0005; for TBX20 vs. Ile219Arg + TBX20: t = 8.1633, *p* = 0.0001; for Ile219Arg vs. TBX20 + (−): t = 2.5833, *p* = 0.1345; for Ile219Arg vs. Ile219Arg + TBX20: t = 0.8667, *p* = 0.8953; and for TBX20 + (−) vs. Ile219Arg + TBX20: t = 1.7167, *p* = 0.4454.

### 3.5. Decreased Binding Ability of Ile219Arg-Mutant TBX20 to the ANP Promoter

**Figure 5:**
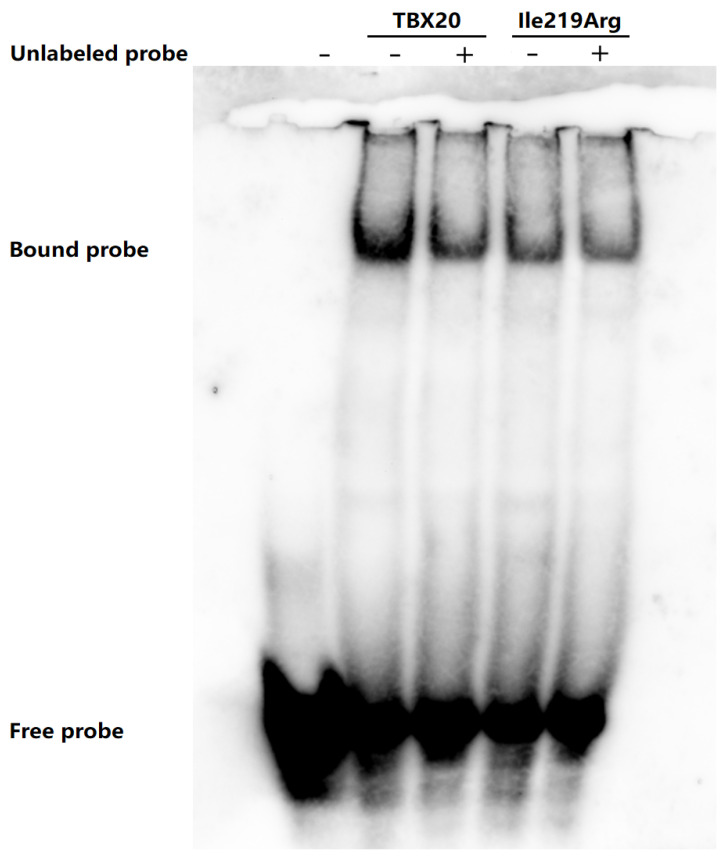
Significantly decreased binding ability of the Ile219Arg-mutant TBX20 to the *ANP* promoter. Wild-type human TBX20 (TBX20) could properly bind to the *ANP* promoter, whereas the DNA-binding ability of Ile219Arg-mutant human TBX20 (Ile219Arg) to the *ANP* promoter was significantly reduced.

As presented in Figure 5, electrophoretic mobility shift assays indicated that the nuclear extracts of the COS7 cells expressing wild-type human TBX20 (TBX20) could interact properly with the *ANP* promoter probe to generate normal DNA-TBX20 complexes. In contrast to that of TBX20, the binding ability of the Ile219Arg-mutant human TBX20 (Ile219Arg) to the *ANP* promoter DNA probe was significantly reduced.

### 3.6. Abnormal Subcellular Localization of Ile219Arg-Mutant TBX20

**Figure 6:**
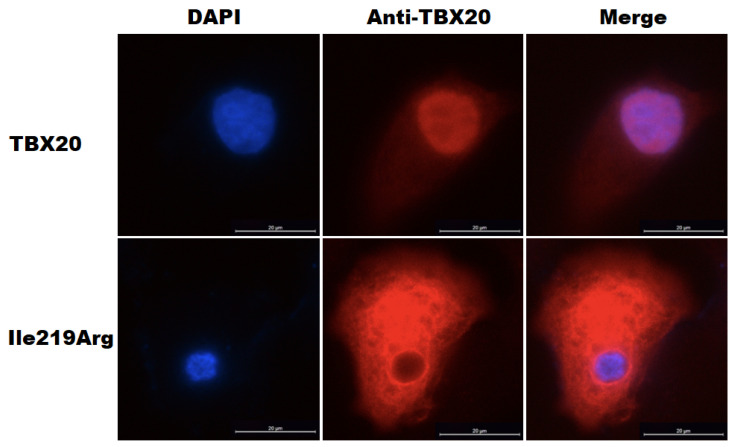
Abnormal intracellular localization of the Ile219Arg-mutant TBX20. Subcellular localization of the wild-type human TBX20 (TBX20) and Ile219Arg-mutant human TBX20 (Ile219Arg) in cultured COS7 cells was observed under a confocal fluorescence microscope. The COS7 cells expressing TBX20 or Ile219Arg were stained using anti-TBX20 and DAPI. The magnified cellular fluorescent images showed that, unlike TBX20, Ile219Arg had an abnormal subcellular distribution, predominantly in the cytoplasm rather than the cellular nuclei.

As exhibited in Figure 6, wild-type human TBX20 (TBX20) was normally localized predominantly within the cellular nucleus. Unlike TBX20, Ile219Arg-mutant human TBX20 (Ile219Arg) showed an abnormal subcellular localization, with a major distribution to the cytoplasm rather than the cellular nucleus.

## 4. Discussion

In the current research, a new locus linked to congenital BAV was located at human chromosome 7p14 by a genome-wide screening with microsatellite markers, linkage assays, and haplotype ascertainments in a pedigree affected with congenital BAV. A WES analysis followed by a Sanger sequencing examination uncovered that, with the located BAV-causing locus, only the *TBX20* c.656T>G (p.Ile219Arg) mutation was in co-segregation with the BAV phenotype in the entire pedigree (Family BAV-1). The missense *TBX20* mutation was absent from the 322 healthy subjects employed as controls and was not released from the databases of SNP and gnomAD. Biochemical analyses unraveled that the Ile219Arg-mutant TBX20 had a significantly diminished transactivation of its representative target gene *ANP* and reduced DNA-binding ability to the promoter of *ANP*, which might be attributed, at least in part, to decreased nuclear distribution of total TBX20 proteins. Hence, these results map a new familial BAV-causative locus at the human chromosome 7p14 and suggest that genetically defective *TBX20* predisposes to congenital BAV.

The *TBX20* gene, which belongs to an important member of an ancient T-Box gene superfamily, maps to human chromosome 7p14–p15, encoding a transcription factor protein that consists of 447 amino acids [27,38]. During embryogenesis, the ample expression of TBX20 is observed in the heart throughout evolution from arthropod to vertebrate and in almost all cardiogenic cell lineages from both cardiogenic heart fields, indicating that TBX20 serves as a key player in regulating the proper development of the cardiovascular system [38,39,40,41,42,43,44,45]. Importantly, recent investigations have substantiated that TBX20 exerts a fundamental role in normal heart valve development [46,47,48,49,50]. In endocardial cushion cells cultured in vitro, the overexpression of *TBX20* was associated with increased cushion mesenchyme proliferation, augmented expression of MMP13 and MMP9, and decreased chondroitin sulfate proteoglycans, encompassing versican and aggrecan; in contrast, the knockdown of *TBX20* was associated with decreased cushion mesenchyme proliferation, lessened expression of MMP13 and MMP9, and increased chondroitin sulfate proteoglycans, supporting that during embryogenesis, TBX20 has a key role in mediating cell proliferation and extracellular matrix remodeling in the precursor populations of mesenchymal valve in endocardial cushions [46]. In addition, treatment with BMP2 increased the expression of TBX20 in the cells from endocardial cushions, and the lack of TBX20 enhanced the expression of TBX2 and diminished the expression of NMYC1 [46]. In mice, the deletion of *Tbx20* in the cardiac atrioventricular canal myocardium led to failure in the constriction of the atrioventricular canal, and the endocardial EMT was severely perturbed [47]. In addition, in the atrioventricular canal myocardium, *Tbx20* was demonstrated to have a role in maintaining the expression and function of multiple genes, including *Bmp2* and *Tbx3*, and the *Bmp2* downstream genes related to the initiation of EMT were also downregulated [47]. Furthermore, the re-expression of *Bmp2* in the myocardium of the atrioventricular canal substantially rescued the defects of EMT caused by the lack of *Tbx20*, indicating that *Bmp2* is one of the key target genes of TBX20 in the development of the cardiac atrioventricular canal [47]. Additionally, *Tbx20* has been defined as a direct transcriptional target of the BMP/SMAD (BMP2/SMAD1) signaling pathway, exerting a pivotal role in the normal morphogenesis of the atrioventricular canal and outflow tract [48,49]. On the other hand, TBX20 was found to directly interfere with the Bmp/Smad signaling pathway to suppress the expression of TBX2 in the developing chambers, thereby confining the expression of TBX2 to the region of the atrioventricular canal [50]. Moreover, TBX20 was verified to be highly expressed in the heart valves and endocardial cushions throughout cardiac organogenesis, and in mice, the knockout of *Tbx20* in endocardial cells led to severe defects in valve elongation and maturation (mainly due to aberrant endocardial cell proliferation and extracellular matrix development in valves), impaired cardiac function, and an abnormal Wnt/β-catenin signaling pathway in the endocardial cushions [51]. Collectively, these findings demonstrate that TBX20 plays a wide variety of fundamental roles in cardiovascular development, especially in cardiac valvular morphogenesis, and genetically compromised *TBX20* predisposes to anomalous cardiac valvulogenesis, including BAV.

Previous multiple investigations have validated that TBX20 transactivates the expression of a wide variety of downstream target genes expressed abundantly in the heart, encompassing *ANP*, *KCNH2*, *GJC1*, and *GJA5*, alone or synergistically with transcriptionally cooperative partners, encompassing NKX2.5, GATA5, GATA4, and TBX5 [27,52,53,54], and malicious variations in the genes *NKX2.5* [55], *GATA5* [56,57,58], *GATA4* [59,60], and *TBX5* [61] have been implicated with the pathogenesis of congenital BAV in humans. In this research, a new *TBX20* mutation was discovered to be accountable for familial BAV. These observational results provide genetic evidence favoring that dysfunctional *TBX20* contributes to BAV, presumably by decreasing the expression of several key target genes.

Recently, *TBX20* variations have been involved in sporadic congenital BAV [62]. Luyckx and colleagues [62] performed a rare variation burden assay utilizing the sequence data of 637 cases affected with congenital BAV/thoracic aortic aneurysm for two (*TBX20* and *DGCR6*) of the seven candidate genes carrying initially verified copy number variations (duplications and/or deletions), which revealed a suggestive genetic role for *TBX20* in the etiology for BAV/TAA. As a result, four rare deleterious *TBX20* variants were identified in the seven unrelated patients with congenital BAV/thoracic aortic aneurysm, including p.Ile39Met (c.117C>G) in one patient, p.Ser125* (c.374C>A) in one patient, p.Asp176Ala (c.527A>C) in four unrelated patients, and p.Pro178Leu (c.533C>T) in one patient. Except for p.Pro178Leu (c.533C>T), the other three *TBX20* variants were absent from the gnomAD database [62]. This study suggests that deleterious *TBX20* variants predispose to BAV/thoracic aortic aneurysm, though the functional effects of these variations remain to be expounded.

The crucial role of *TBX20* in the pathogenesis of CHD has been extensively explored [63,64,65,66,67,68,69,70,71,72]. In 2007, Kirk and coworkers [63] first reported two missense mutations and one nonsense mutation in *TBX20* identified in patients suffering from diverse cardiac pathologies, encompassing defects of septation (atrial/ventricular septal defect) and valvular malformation (mitral valve stenosis and prolapse), aortic coarctation, and cardiomyopathy [63]. Subsequently, *TBX20* mutational screening investigations expanded the clinical phenotypic spectrum linked to deleterious *TBX20* mutations, including the double-outlet right ventricle, Fallot’s tetralogy/pentalogy, patent ductus arteriosus, common atrioventricular canals, aortic coarctation, and persistent truncus arteriosus, and these investigations unveiled more *TBX20* mutations contributing to CHD [64,65,66,67]. In addition, *TBX20* mutations have been discovered to contribute to dilated cardiomyopathy and/or left ventricular noncompaction [68,69,70,71,72], as well as atrial fibrillation [27]. In the current investigation, in addition to congenital BAV, three patients carrying a *TBX20* loss-of-function mutation (Family BAV-1: II-9, III-17, and IV-12) also had congenital ASD. These studies highlight the integral roles of *TBX20* in normal cardiovascular morphogenesis and postnatal cardiac homeostasis, as well as structural remodeling, suggesting that genetically defective *TBX20* predisposes to distinct heart diseases, including CHD, dilated cardiomyopathy, and arrhythmia.

There are some potential limitations to the present research. Firstly, the change in luciferase activity can be influenced by the amount of protein produced. Although the amount of DNA theoretically predicts the amount of protein and a dual-luciferase reporter assay could normalize the transfection efficiency, it is best to provide Western blot data, confirming that the wild-type and mutant proteins are expressed in equivalent amounts and that changes in DNA input produce corresponding changes in protein levels. Secondly, although the molar excess of a cold competing probe was used and the cold competitor significantly blocked the specific DNA–protein binding, it did not seem to block the specific signal completely as expected. A larger dose of the cold competing probe should be used to block the specific signal completely. Thirdly, considering the loss of nuclear import of the mutant TBX20 protein, the slight decrease in the EMSA signal of the mutant may be due to a lower level of nuclear mutant TBX20 proteins. To rule out this possibility, a complementary Western blot of the nuclear extracts would be helpful. Fourthly, analyzing the effect of mutant TBX20 on other target genes will provide more insight into the pathogenic mechanism. Finally, the establishment of a gene knock-in animal model expressing the Ile219Arg-mutant TBX20 protein will provide a practical tool to investigate the pathogenicity of the identified *TBX20* mutation.

## 5. Conclusions

The present investigation maps a new familial BAV-causing locus to human chromosome 7p14 and uncovers *TBX20* as a familial BAV-causative gene, adding insight into the molecular pathogenesis underlying congenital familial BAV and providing a new molecular target for the potential personalized therapies for familial BAV.

## Figures and Tables

**Table 1:** Clinical characteristic information and status of the TBX20 Ile219Arg mutation of the living pedigree members with bicuspid aortic valve from Family BAV-1.

Identity (Family BAV-1)	Sex	Ages (Years)	Phenotypes (Structural Heart Defects)	Genotypes (TBX20 Ile219Arg Mutation)
II-1	Male	69	BAV, AVS	+/−
II-6	Female	64	BAV, AVS	+/−
II-9	Male	60	BAV, AVS, ASD	+/−
II-12	Female	53	BAV	+/−
III-2	Female	45	BAV	+/−
III-5	Male	40	BAV	+/−
III-10	Female	39	BAV	+/−
III-17	Male	35	BAV, ASD	+/−
III-21	Male	23	BAV	+/−
IV-2	Male	20	BAV	+/−
IV-4	Female	17	BAV	+/−
IV-8	Male	16	BAV	+/−
IV-12	Male	10	BAV, ASD	+/−

ASD: atrial septal defect; AVS: aortic valve stenosis; BAV: bicuspid aortic valve; +/−: heterozygote.

**Table 2:** The scores of two-point logarithms of odds for the chosen eight genetic markers at chromosome 7p14.

Marker	Scores of Two-Point Logarithms of Odds at Recombination Fraction θ=						
	0.00	0.01	0.05	0.10	0.20	0.30	0.40
D7S683	(−∞)	0.7466	1.8580	2.0844	1.8682	1.2969	0.5428
D7S656	(−∞)	0.7466	1.8580	2.0844	1.8681	1.2952	0.5212
D7S484	5.2802	5.1929	4.8348	4.3654	3.3440	2.1928	0.9290
D7S2250	5.4185	5.3312	4.9730	4.5034	3.4804	2.3232	1.0247
D7S2251	2.0081	1.9710	1.8201	1.6254	1.2144	0.7774	0.3427
D7S528	4.2144	4.1446	3.8580	3.4823	2.6640	1.7400	0.7339
D7S2497	5.4185	5.3312	4.9730	4.5034	3.4804	2.3232	1.0242
D7S2507	(−∞)	1.2590	1.7430	1.7622	1.4497	0.9355	0.3356

## Data Availability

All data are provided in this manuscript, along with Appendix A Table A1.

## Abbreviations

 The following abbreviations are used in this manuscript:

ANNOVAR	Annotation of variation
ANP	Atrial natriuretic peptide
ASD	Arial septal defect
BSA	Bovine serum albumin
BWA	Burrows–Wheeler alignment
CHD	Congenital heart disease
DAPI	4′,6-Diamidino-2-phenlindole
DOAJ	Directory of Open Access Journals
EMT	Endothelial-to-mesenchymal transition
GATK	Genome analysis toolkit
gDNA	Genomic DNA
gnomAD	Genome aggregation database
LD	Linear dichroism
LOD	Logarithms of odds
Luc	Luciferase
MDPI	Multidisciplinary Digital Publishing Institute
PBS	Phosphate buffer saline
PCR	Polymerase chain reaction
SD	Standard deviation
SHF	Second heart field
SNP	Single-nucleotide polymorphism
TBX20	T-box 20
TLA	Three-letter acronym
VEP	Variant effect predictor
WES	Whole-exome sequencing

## Appendix A

**Table A1:** A list of all 87 genes at the human chromosomal locus between D7S656 and D7S2507.

CFAP144P1 (pseudo)	KRT8P20 (pseudo)	**AMPH**	RN7SL83P (pseudo)	TRG-AS1 (lncRNA)
TRG (pseudo)	TRGV1 (pseudo)	TRGV2 (pseudo)	TRGV3 (pseudo)	TRGV4 (pseudo)
TRGV5 (pseudo)	TRGV5P (pseudo)	TRGV6 (pseudo)	TRGV7 (pseudo)	TRGV8 (pseudo)
TRGVA (pseudo)	TRGV9 (pseudo)	LOC103689914 (pseudo)	TRGV10 (pseudo)	TRGVB (pseudo)
TRGV11 (pseudo)	TRGJP1 (pseudo)	TRGJP (pseudo)	**TARP**	TRGJ1 (pseudo)
TRGC1 (pseudo)	TRGJP2 (pseudo)	TRGJ2 (pseudo)	TRGC2 (pseudo)	**STARD3NL**
LOC105375236 (lncRNA)	**EPDR1**	**SFRP4**	**NME8**	CDCA3P1 (pseudo)
GPR141BP (pseudo)	**GPR141**	NECAP1P1 (pseudo)	**ELMO1**	LOC124900242 (pseudo)
RPS10P14 (pseudo)	RNU6-565P (pseudo)	LOC124901833 (lncRNA)	RPS17P13 (pseudo)	ELMO1-AS1 (lncRNA)
MIR1200 (miRNA)	LOC105375235 (lncRNA)	NPM1P18 (pseudo)	**AOAH**	AOAH-IT1 (lncRNA)
**ANLN**	**MATCAP2**	**EEPD1**	MARK2P7 (pseudo)	MARK2P13 (pseudo)
LOC101928618 (lncRNA)	LOC105375233 (lncRNA)	LOC107986734 (lncRNA)	SEPTIN7P3 (pseudo)	PPP1R14BP4 (pseudo)
LOC100419774 (pseudo)	RNU6-1085P (pseudo)	**SEPTIN7**	LOC107986783 (pseudo)	SEPTIN7-DT (lncRNA)
LOC442293 (pseudo)	LOC124901615 (lncRNA)	LINC03013 (lncRNA)	**HERPUD2**	HERPUD2-AS1 (lncRNA)
LOC101928421 (lncRNA)	LOC105375230 (lncRNA)	LOC401324 (lncRNA)	**TBX20**	DPY19L2P1 (pseudo)
S100A11P2 (pseudo)	LOC105375228 (lncRNA)	**DPY19L1**	LOC102724723 (lncRNA)	MIR548N (miRNA)
**NPSR1**	NPSR1-AS1 (lncRNA)	RN7SL132P (pseudo)	NCAPD2P1 (pseudo)	RPL7P31 (pseudo)
RNU6-438P (pseudo)	**BMPER**			

The genes coding for proteins are shown in bold fonts.
